# A Potential Pitfall in POCUS of the Gallbladder: Beware of the Duodenum

**DOI:** 10.24908/pocus.v7i2.15632

**Published:** 2022-11-21

**Authors:** Fan J Yang, Brian Kohen, Sowmya Sanapala, Michael Halperin

**Affiliations:** 1 Jacobi Medical Center Bronx, New York USA; 2 Albert Einstein College of Medicine Bronx, New York USA; 3 Memorial Regional Hospital Hollywood, Florida USA; 4 New York Presbyterian- Cornell New York, New York USA

**Keywords:** Abdominal point-of-care ultrasound, mimic, acute cholecystitis, duodenum, false positive, gallbladder

## Abstract

It is estimated that 20 million people in the United States have gallbladder disease. Of the patients who present to the Emergency Department (ED) with abdominal pain, 3-10% have acute cholecystitis. Point-of-care ultrasound (POCUS) evaluation of the biliary system is a valuable tool to diagnose gallbladder disease and can greatly expedite the diagnostic evaluation of patients. One source of error in POCUS of the gallbladder is imaging nearby structures that can mimic the gallbladder, such as the duodenum.

## Presentation and discussion 

**Figure 1:**
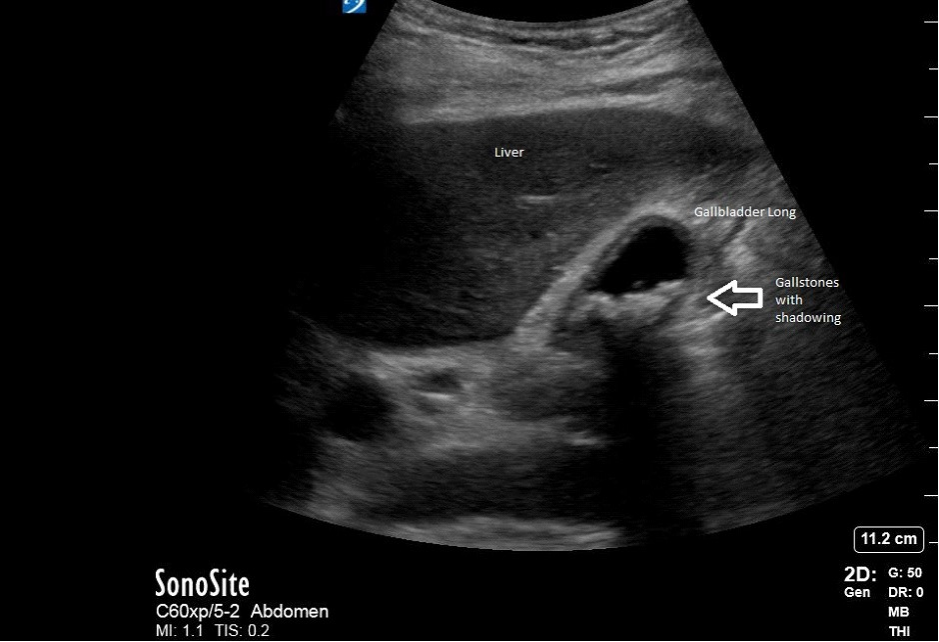
Gallbladder POCUS concerning for acute cholecystitis: gallbladder with thickened anterior wall, shadowing gallstones, and a positive sonographic Murphy sign.

A middle-aged woman with a past medical history of hypertension, end stage renal disease, and human immunodeficiency virus infection presented with persistent and progressively worsening epigastric abdominal pain for one week. She had fevers and chills associated with episodes of non-bloody, non-biliary emesis. On arrival, her vital signs were significant for a blood pressure of 196/102 mmHg, heart rate of 127 bpm, respiratory rate of 22 bpm and temperature of 100.4 °F. A gallbladder POCUS examination was concerning for acute cholecystitis, given the presence of shadowing gallstones, anterior gallbladder wall thickening and a positive sonographic Murphy sign (Figure 1). The patient was fluid resuscitated, received intravenous (IV) antibiotics and pain medication. The surgery service was consulted for cholecystectomy. 

**Figure 2:**
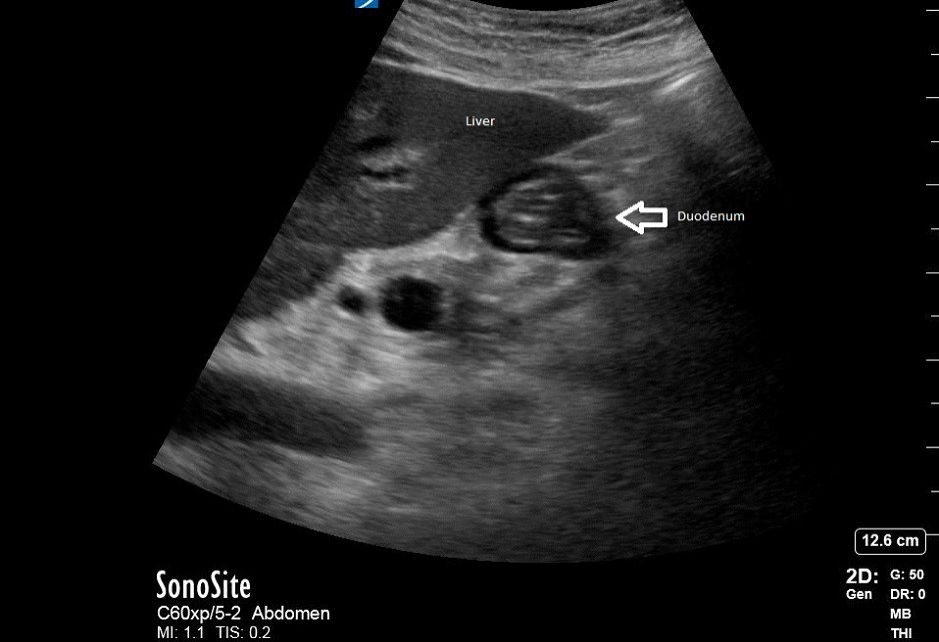
Repeat POCUS Examination with arrow pointing towards the duodenum, a common mimic of the gallbladder.

The patient then began complaining of increasing pain despite IV opioid analgesia. The emergency medicine provider repeated a POCUS of the gallbladder. At this point, the clinician believed the gallbladder looked different. It appeared to contain heterogeneous material surrounded by simple anechoic fluid (Figure 2). These findings were thought to be new pericholecystic fluid, concerning for progression of the acute cholecystitis, to possibly gallbladder perforation. The clinician sought out a colleague who correctly identified this structure as the duodenum, a known gallbladder mimic (Figure 2). 

These two examinations performed on the same patient demonstrate a potential pitfall in POCUS of the gallbladder: the duodenum can mimic the gallbladder in appearance and lead to erroneous conclusions 1. In this case, the stool and fluid-filled duodenum on repeat POCUS was initially falsely interpreted as a gallbladder with pericholecystic fluid. A follow-up comprehensive hepatobiliary ultrasound confirmed the finding of acute cholecystitis and the patient was admitted for surgery. 

## Conclusion

Acute cholecystitis, a common diagnosis in the ED, requires emergent surgery, with earlier cholecystectomy time yielding improved outcomes, shorter hospital stay, and decreased healthcare costs 1, 2. However, during diagnostic workup, false positive rates can be seen in up to 16% comprehensive radiographic ultrasounds. The most common mimic which can lead to a false positive is an air-filled duodenum 3, 4. Other gallbladder mimics include renal or hepatic cysts, hepatic vessels, and the inferior vena cava. Therefore, it is critical for providers performing POCUS biliary exams to be cognisant of these mimics so the proper diagnosis can be made. 

In this case, diagnostic evidence of both true and false positive findings of acute cholecystitis were seen. A side-by-side comparison video also highlights the ultrasonographic differences seen in the POCUS biliary exam (online Video S1). 

It is critical that POCUS of the gallbladder include multiple views to avoid such structures that mimic the gallbladder. Additionally, knowledge and identification of the sonographic landmarks for the gallbladder such as the main lobar fissure and the portal triad may help avoid these common mimics. In the hands of a skilled emergency medicine provider, POCUS can shorten ED visit time, lead to earlier diagnosis, expedite treatment and, ultimately, improve patient centered care 5. 

## Consent/Ethics

Informed consent was obtained from the patient by the authors. The patient has consented to the use of de-identified images, video clips, and health information to be published within the journal. 

## Disclosures

None.

## Supplementary Material

 Video S1 Video showing side by side clips of patient’s initial and then follow up POCUS examination.
